# Supplementary material for: Anionic redox reaction in layered NaCr2/3Ti1/3S2 through electron holes formation and dimerization of S–S
Source: Nat Commun. 2019 Oct 1;10:4458. doi: 10.1038/s41467-019-12310-6 (PMC6773774; doi:10.1038/s41467-019-12310-6)
Supplement: Supplementary file 1 — Supplementary Information [file 41467_2019_12310_MOESM1_ESM.pdf]

Supplementary Information

Anionic redox reaction in layered $NaCr_{2/3}Ti_{1/3}S_2$ through electron holes formation and dimerization of S-S

Tian Wang[1]†, Guo-Xi Ren[2,9,11]†, Zulipiya Shadike[3]†, Ji-Li Yue[4], Ming-Hui Cao[1], Jie-Nan Zhang[5], Ming-Wei Chen[6], Xiao-Qing Yang[3], Seong-Min Bak[3], Paul Northrup[10], Pan Liu[6] *, Xiao-Song Liu[2,7,8,11] * & Zheng-Wen Fu[1] *

1 Shanghai Key Laboratory of Molecular Catalysts and Innovative Materials, Department of Chemistry & Laser Chemistry Institute, Fudan University, Shanghai, 200433, China.

2 State Key Laboratory of Functional Materials for Informatics, Shanghai Institute of Microsystem and Information Technology, Chinese Academy of Science, Shanghai 200050, China.

3 Chemistry Division, Brookhaven National Laboratory, Upton, New York, 11973, USA

4 School of Materials Science and Engineering, Herbert Gleiter Institute of Nanoscience, Nanjing University of Science and Technology, Nanjing, Jiangsu 210094, China

5 Beijing National Laboratory for Condensed Matter Physics, Institute of Physics, Chinese Academy of Sciences, Beijing 100190, China.

6 Shanghai Key Laboratory of Advanced High-temperature Materials and Precision Forming, State Key Laboratory of Metal Matrix Composites, School of Materials Science and Engineering, Shanghai Jiao Tong University, Shanghai 200240, China

7 Tianmu Lake Institute of Advanced Energy Storage Technologies, Liyang City, Jiangsu 213300, China

8 School of Physical Science and Technology, ShanghaiTech University, Shanghai 201210, China

9 University of Chinese Academy of Sciences, Beijing 100049, P. R. China.

10 Department of Geosciences, Stony Brook University, Stony Brook, New York, 11794

11 State Key Laboratory of Functional Materials for Informatics；CAS Center for Excellence in Superconducting Electronics（CENSE），Shanghai Institute of Microsystem and Information Technology, Chinese Academy of Sciences, Shanghai 200050, P.R

Tian Wang, Guo-Xi Ren and Zulipiya Shadike contributed equally to this work. Correspondence and requests for materials should be addressed to P.L. (email: panliu@sjtu.edu.cn) or to X.-S.L. (email: xliu3@mail.sim.ac.cn) or to Z.-W.F. (email: zwfu@fudan.edu.cn)

Supplementary Figures

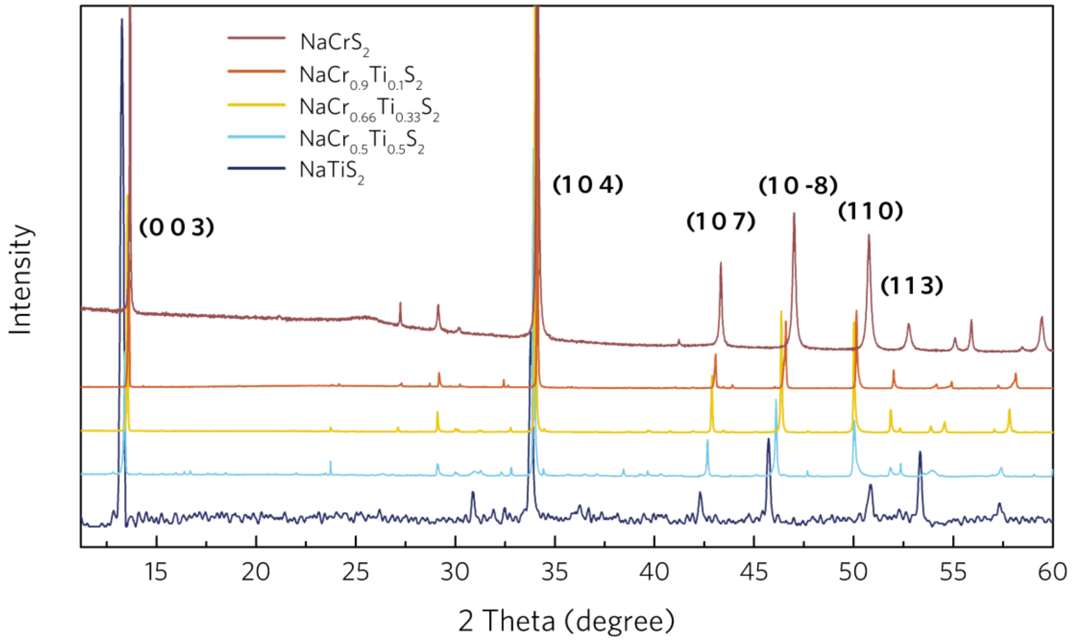

Supplementary Figure 1. XRD patterns of as-prepared $NaCr_xTi_{1-x}S_2$ series particles. The XRD of $NaTiS_2$ was tested by laboratory XRD and others are tested by synchrotron XRD, which wavelengths were adjusted to Cu Ka for comparision. The crystal indices are noted in the figure, the position of peaks move regularly when compositions change. There are impurity peaks for $NaTiS_2$ at 31° and 36°, which can be attributed to oxidized P3 structure Na_xTiS_2 due to the instability of Ti^{3+} and O3-structure material exposed in the air.

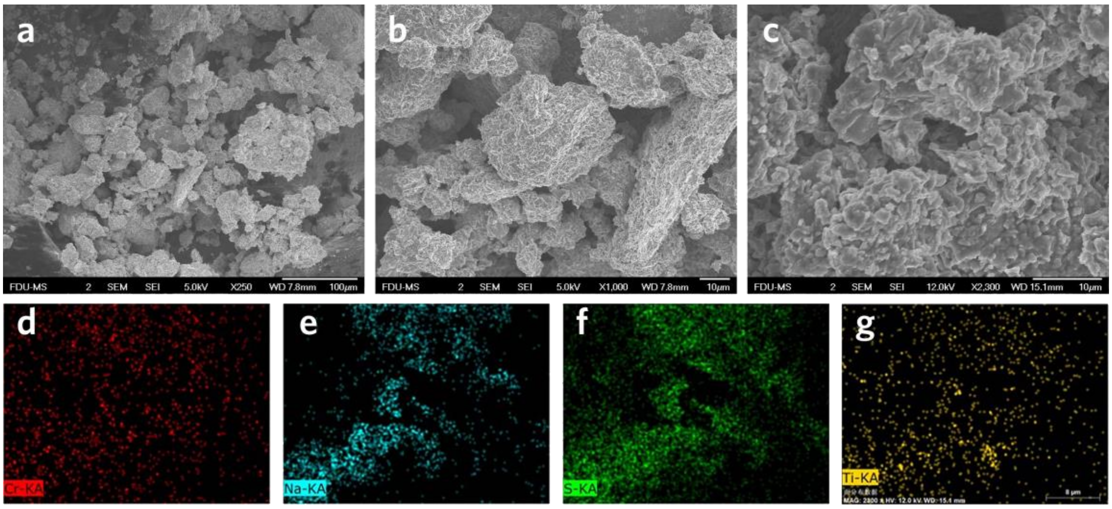

Supplementary Figure 2. SEM images of as-prepared $NaCr_{2/3}Ti_{1/3}S_2$ particles. **a-c,** the SEM images at different scales (scales bars are in the images). **d-g**, the mapping results of Cr, Na, S and Ti tested for Supplementary Figure 1c. The SEM images showed the particles size of material is mostly in the range of 10-100 μm. The mapping results showed that elements distributed uniformly in the interface.

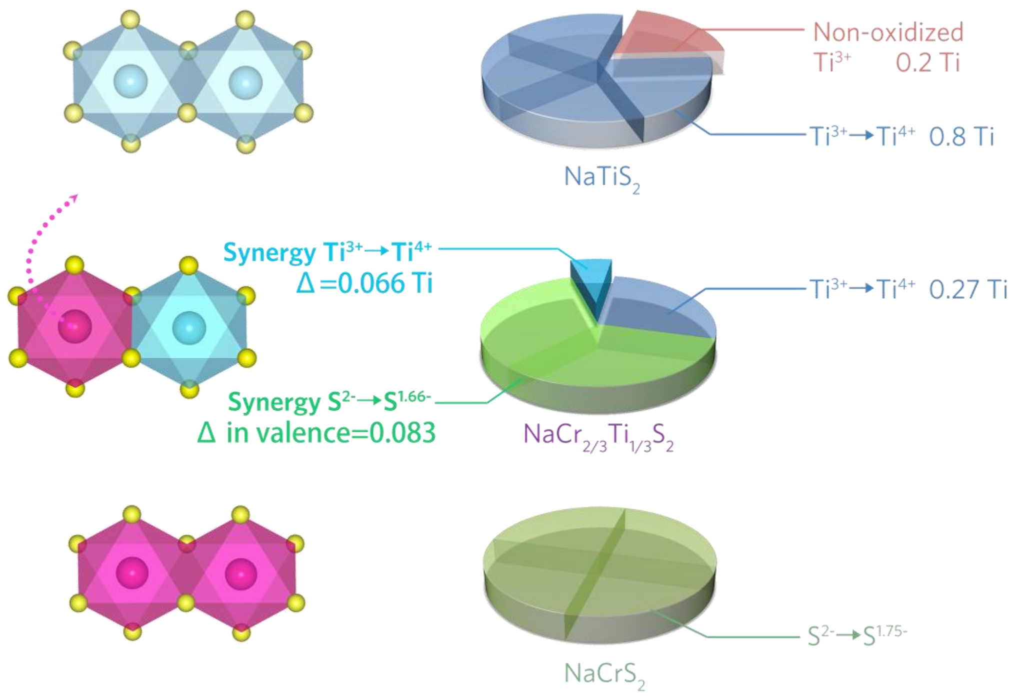

Supplementary Figure 3. **Pie charts representing the ratio of oxidized element in $NaTiS_2$, $NaCr_{2/3}Ti_{1/3}S_2$ and $NaCrS_2$ in the first cycle.** The ratio in pie charts stand for the ratio of element participated in the redox process, i.e. in $NaCrS_2$ it is thought that S^{2-} can only be oxidized to $S^{1.75-}$ in average, and in $NaCr_{2/3}Ti_{1/3}S_2$. S^{2-} can be further oxidized to $S^{1.66-}$ in average. One S chart is in fact standing for 2 times the number of atoms of Ti chart. On the left side are octahedron models of corresponding materials, pink for Cr, blue for Ti and yellow for S, respectively.

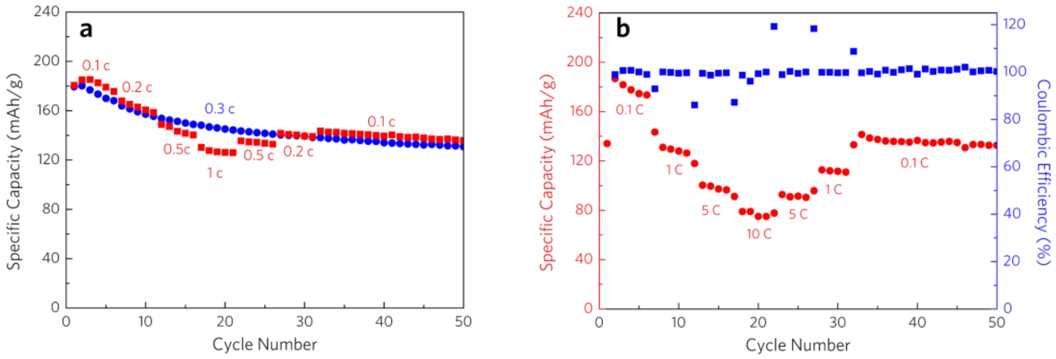

Supplementary Figure 4. Rate performance of $NaCr_{2/3}Ti_{1/3}S_2$ during the first 50 cycles. **a,** the capacity retention of cycling rate between 0.1-1 C versus 0.3 C, to eliminate the effect of capacity loss, the performance under 0.3 C rate was used as contrast. More than tens of coin cells were assembled to test the electrochemical measurements with at least 180±10 mAh/g in the first cycle **b**, the coulumbic efficiency and the capacity between 0.1 C and 10 C. The electrode showed good rate performance and could achieve the capacity of at least 80 mAh/g under 10 C rate

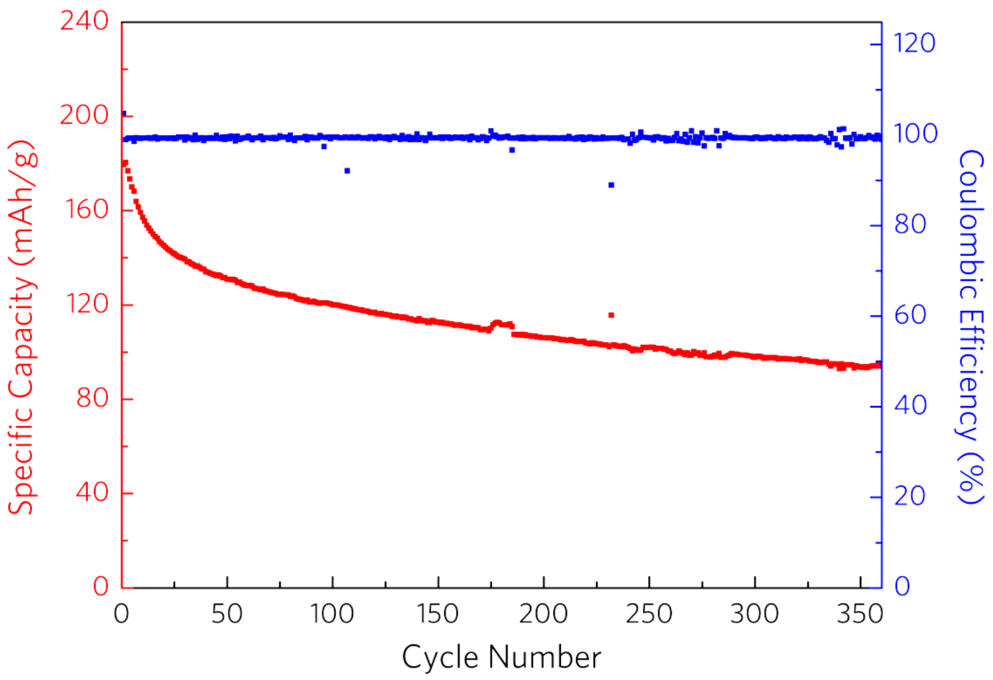

Supplementary Figure 5. Cyclic performance of $NaCr_{2/3}Ti_{1/3}S_2$ during the first 360 cycles at 1/3 C rate. After 360 cycles under 1/3 rate, the coulombic efficiency remains ~100 percent. The capacity retention can reach 52.2 % and the capacity after 360 cycles is 89 mAh/g. The capacity retention is based on the first cycle.

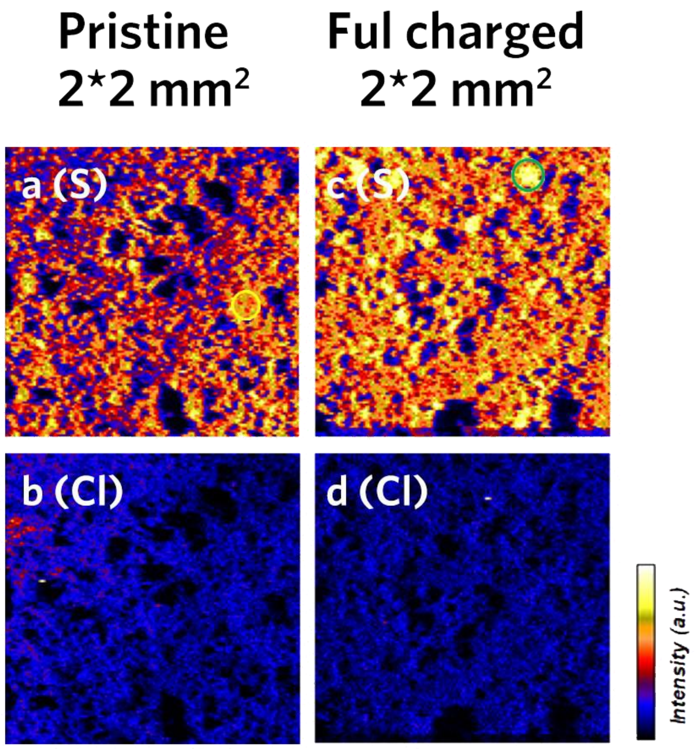

Supplementary Figure 6. 2D scanning XRF images of S and Cl at (**a-b**) the pristine (immersed in the electrolyte) and (**c-d**) fully charged states collected at a energy of 3000 eV. The color of the each pixel in the XRF images reflects the concentration of the S and Cl elements. (dark: low concentration; yellow: high concentration) It can be clearly seen form the XRF images as shown in Figure S6 the sulfur is homogenously distributed in the pristine and charged electrodes. At the same time, the electrolyte surface was covered by Cl, which is from the $NaClO_4$ in the electrolyte. It should be mentioned that, the pristine electrode after immerse in the electrolyte was used for this measurement. To avoid the interference of Cl absorption in the S- K edge XAFS part, a region with high sulfur concentration and low Cl was selected for the S K-edge XAS measurement (yellow and green circle in the Figure S6).

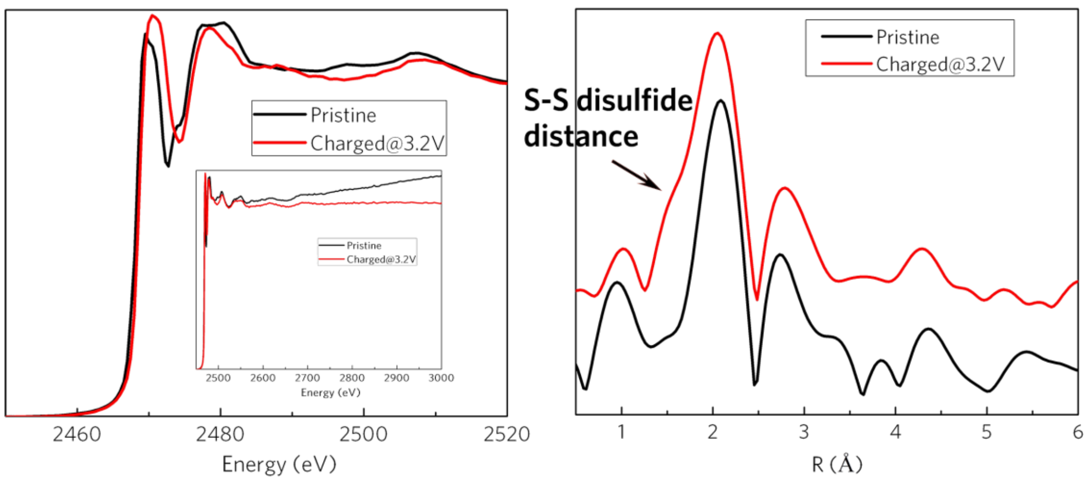

Supplementary Figure 7. Left: sulfur K-edge XANES data for pristine and full charged samples, inset is corresponding sulfur EXAFS data. Right: data converted to R space.

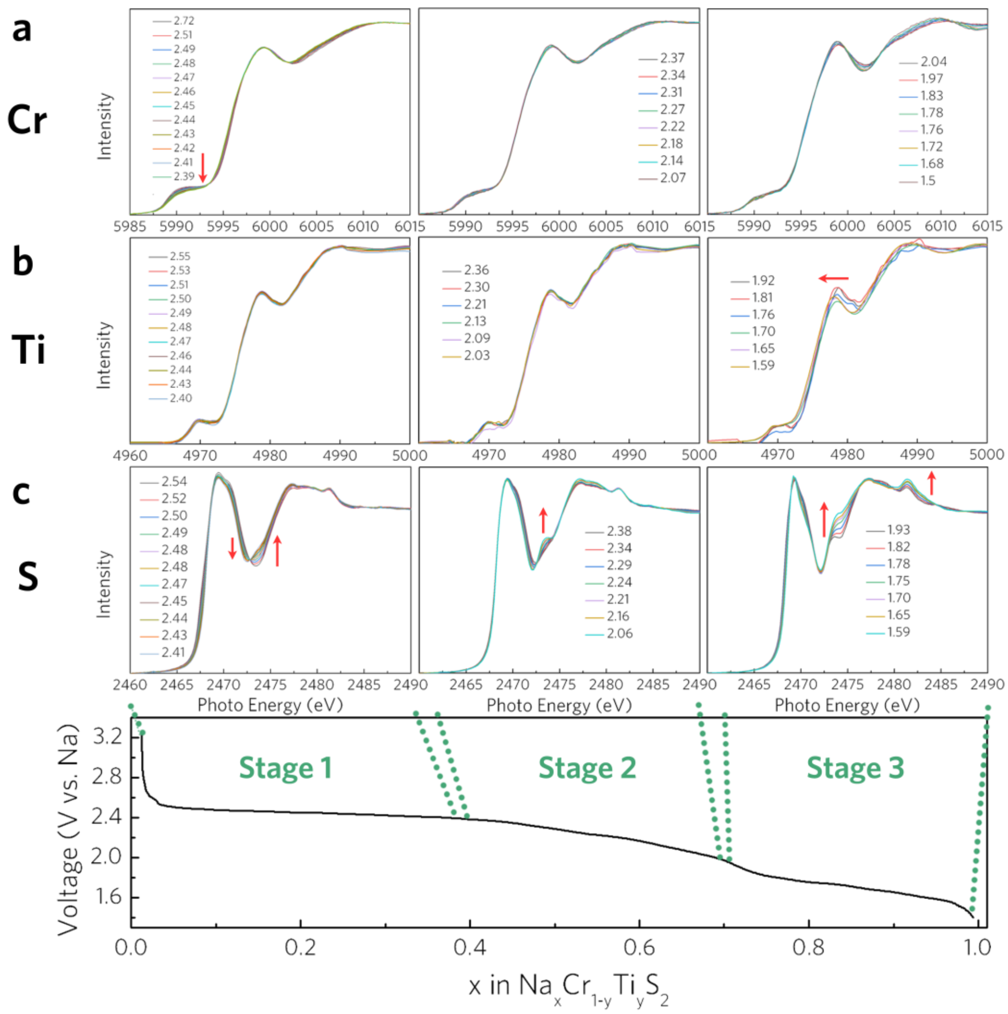

Supplementary Figure 8. In-situ K-edge XAS spectra of **a**. Cr, **b.** Ti and **c.** S valance state, the corresponding voltage profiles for the first discharging process is consisting of three stages is in the bottom. The changes of Ti, S and Cr K edges are complete symmetry with the charging process in the discharging process, and finally return close to those of the pristine material in a symmetrical way, which indicates the reversibility of the charge compensation on Ti and sulfur.

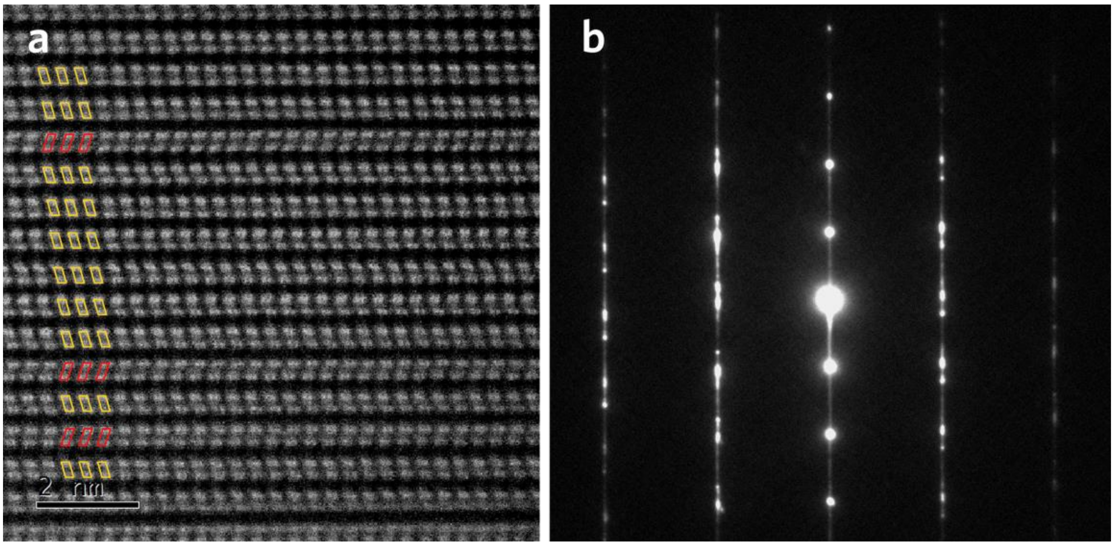

Supplementary Figure 9. STEM image of $Na_{0.5}Cr_{2/3}Ti_{1/3}S_2$ a. STEM pattern of $Na_{0.5}Cr_{2/3}Ti_{1/3}S_2$, built mostly by P3 close packing. The bright spots are S-Cr/Ti-S slabs, with obvious stacking faults, in which the yellow bars stand for P3 stacking and red bars stand for stacking faults. **b.** SAED pattern of $Na_{0.5}Cr_{2/3}Ti_{1/3}S_2$. The result showed a multiphase diffraction pattern.

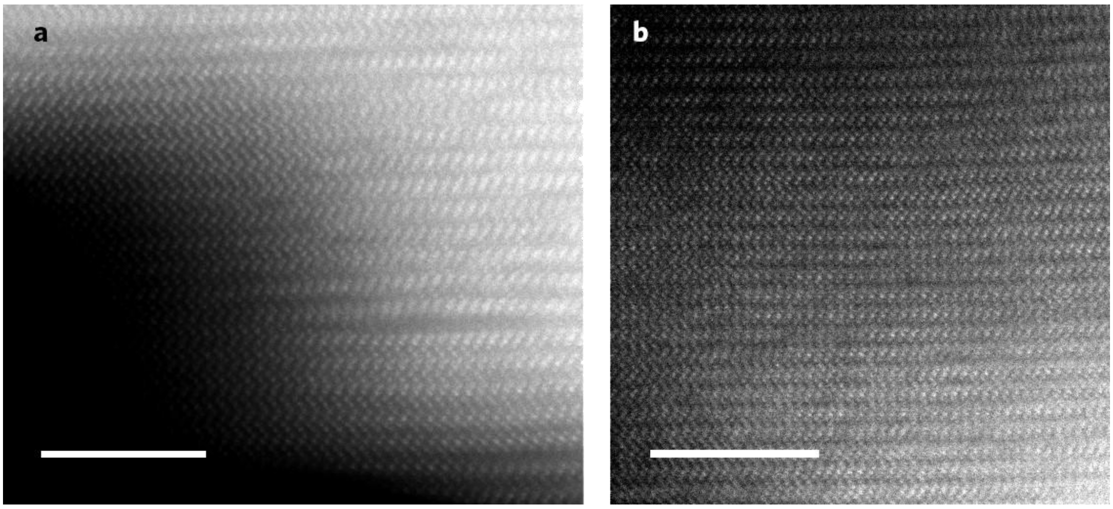

Supplementary Figure 10. HAADF-STEM images of full charged $Na_0Cr_{2/3}Ti_{1/3}S_2$. *scale bar* 5 nm.

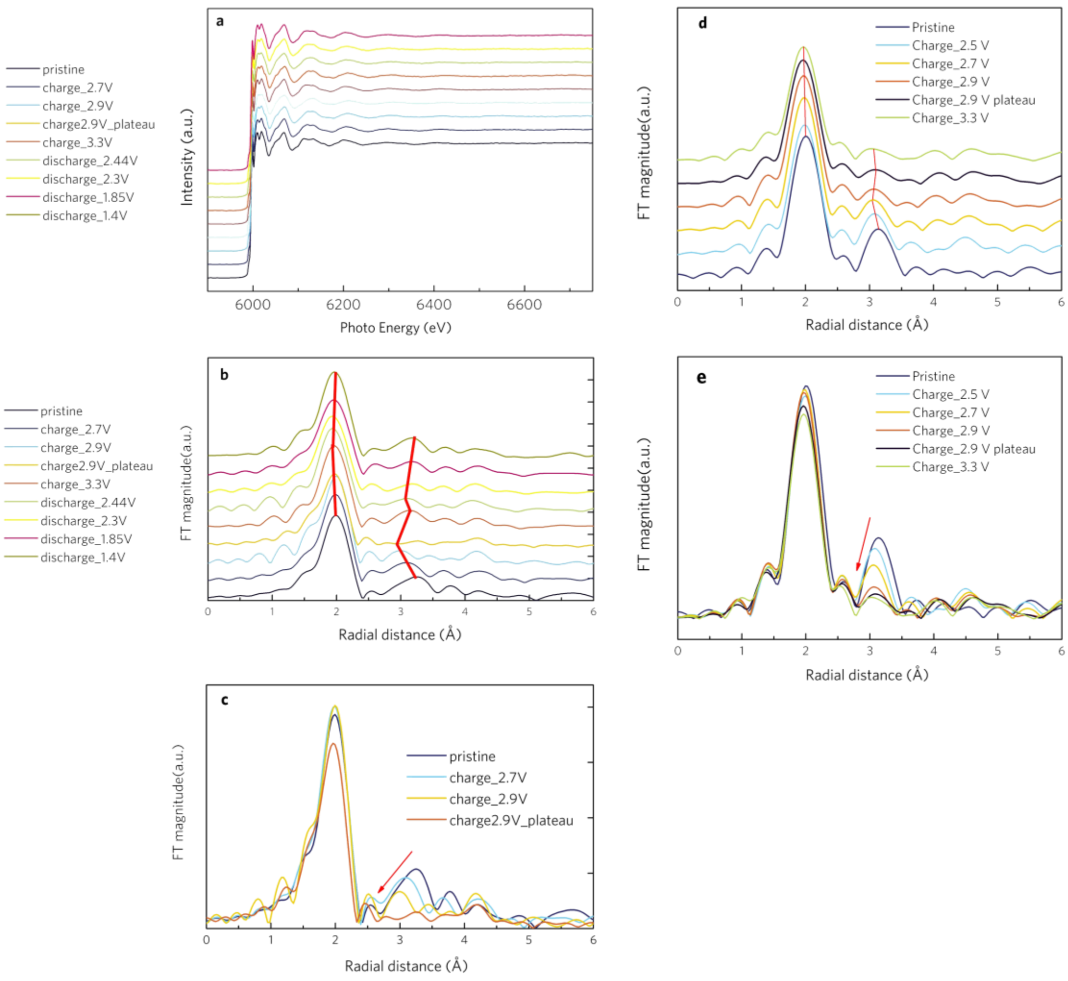

Supplementary Figure 11. a. *Ex situ* Cr K-edge EXAFS profile of $NaCr_{2/3}Ti_{1/3}S_2$ during the first cycle and **b, c.** converted to R space. **d,e.** In situ Cr EXAFS profile of $NaCr_{2/3}Ti_{1/3}S_2$ during the first charging process. The Cr-M peaks intensity at ~3.2 Å is descending in the charging process, indicating the migration of Cr atoms.

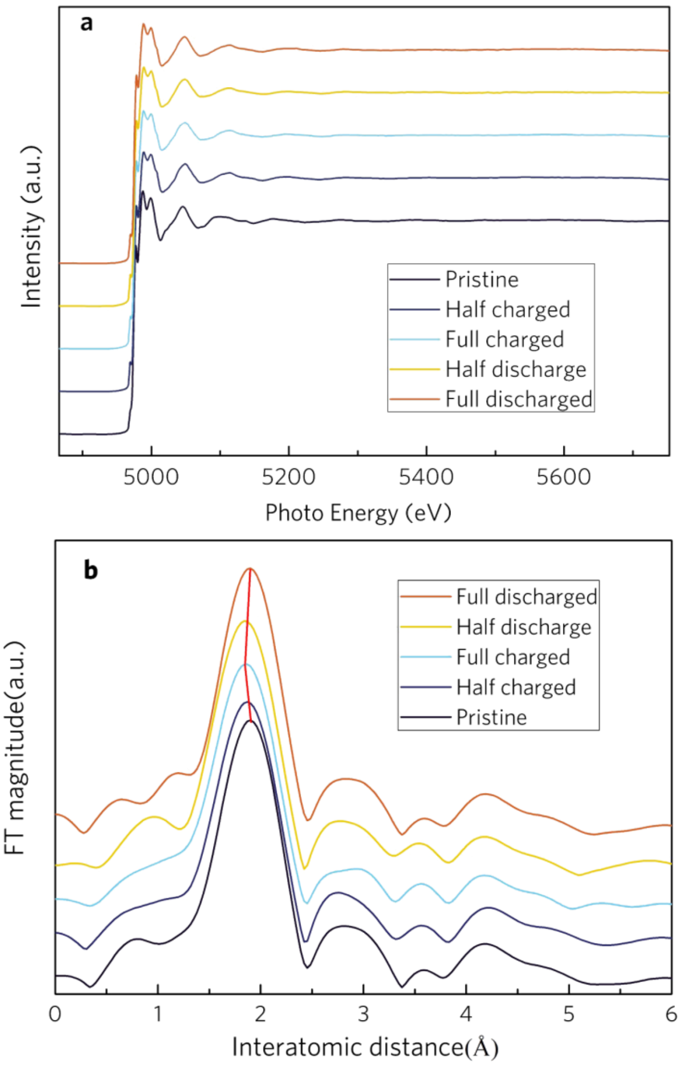

Supplementary Figure 12. a.*Ex situ* Ti K-edge EXAFS profile of $NaCr_{2/3}Ti_{1/3}S_2$ during the first cycle and **b.** converted to R space. The first peak representing Ti-S distance decreases in the charging process and increases in the discharging process, similar to Cr-S. But the intensity of Ti-(Ti/Cr) shell peak is quite low, making it difficult to be further analyzed, probably due to the low amount of Ti relative to Cr.

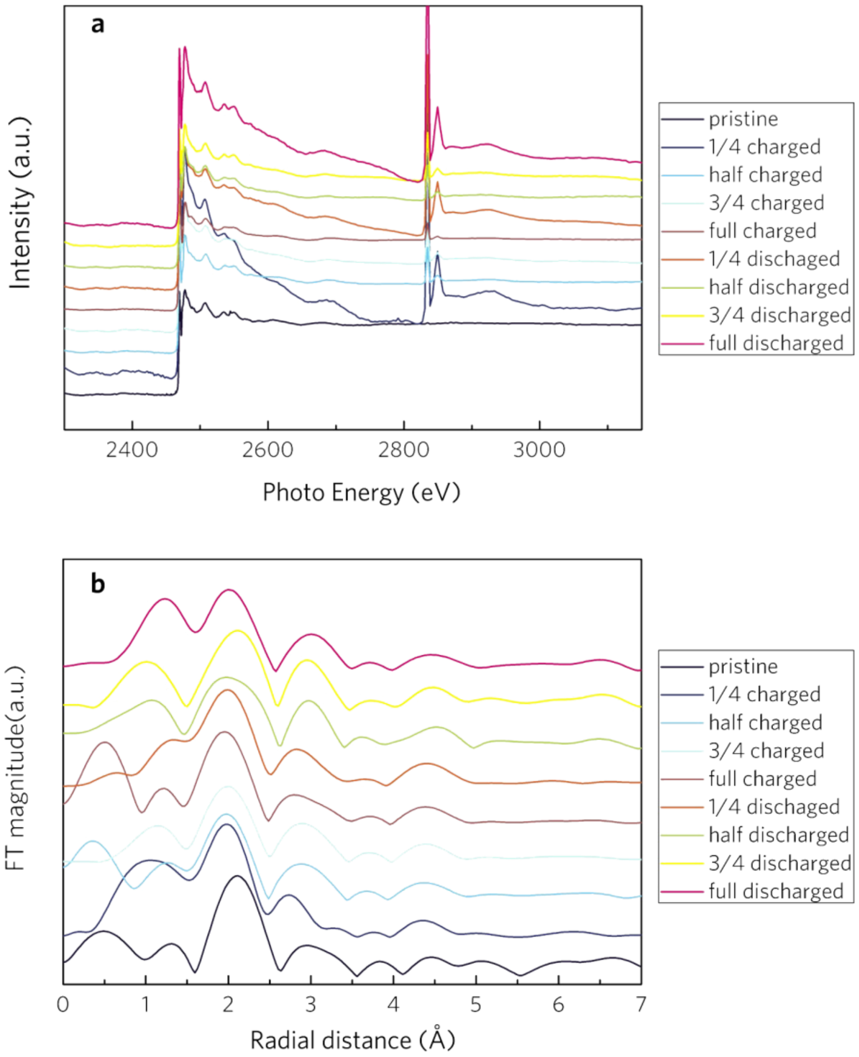

Supplementary Figure 13. a. *Ex situ* S K-edge EXAFS profile of $NaCr_{2/3}Ti_{1/3}S_2$

during the first cycle and **b.** converted to R space. It can be noticed that there is an

obvious peak at ~2850 eV, corresponding to Cl in NaClO4 electrolyte. No Cl peak can be observed in the pristine sample because it hasn't been soaked in NaClO4 electrolyte. The other electrode samples for EXAFS test have been cleansed by DMC for several times, but it seems impossible to clear all the remaining ClO4-, making the available range of S EXAFS shrinking to 2500-2800 eV, which is not enough to export reasonable R space profile.

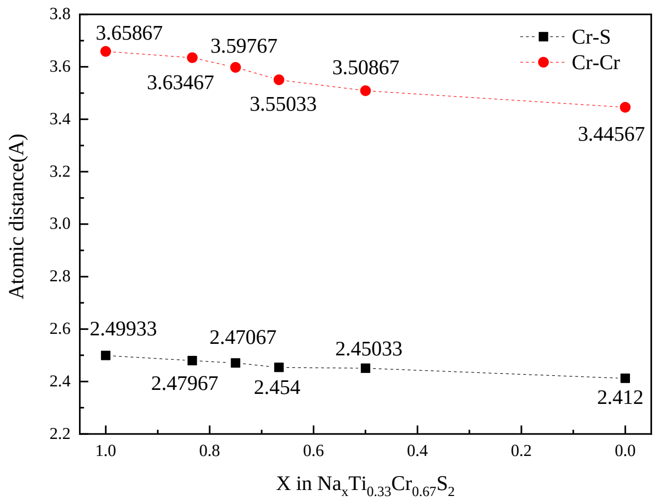

Supplementary Figure 14. Calculated Cr-S and Cr-Cr distance in the first charging process of $NaCr_{2/3}Ti_{1/3}S_2$.

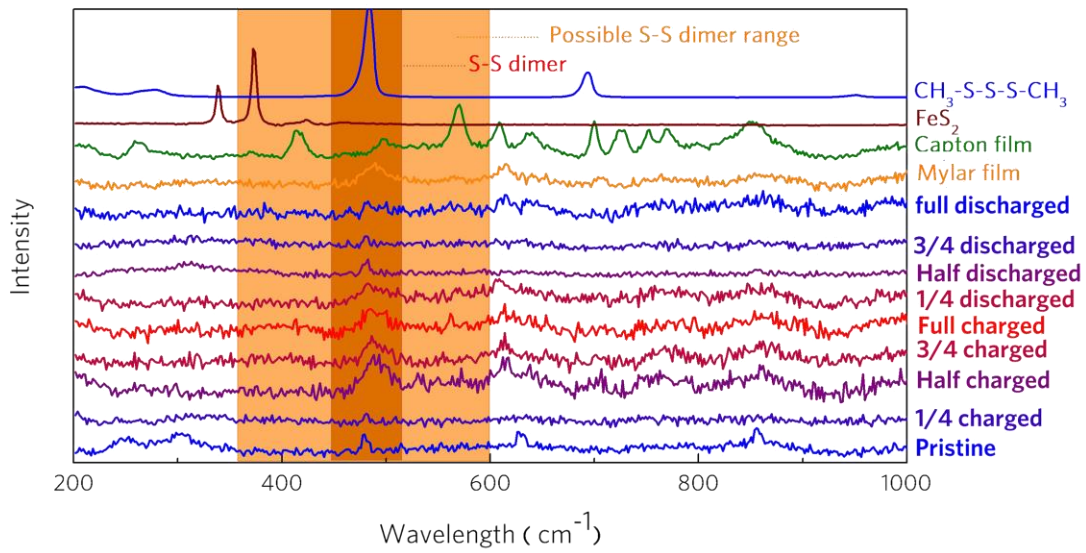

Supplementary Figure 15. Ex situ Raman spectrum data of $NaCr_{2/3}Ti_{1/3}S_2$ during the first cycle versus FeS_2, CH_3-S-S-S-CH_3, mylar film and capton film. 1# stands for the pristine, 5# for the full charged sample and 9# for the full discharged sample. The intervals are divided equally according to capacity. The Capton film was used instead of regular Mylar film to estimate the Raman peak of the sealing films. In this figure, the intensity of peaks may not mean much as they come from *ex situ* tests. The longitudinal distribution of ex situ sample is not uniform, the surface part might be ahead of the average charging process, which would lead to differences in peak intensity as well.

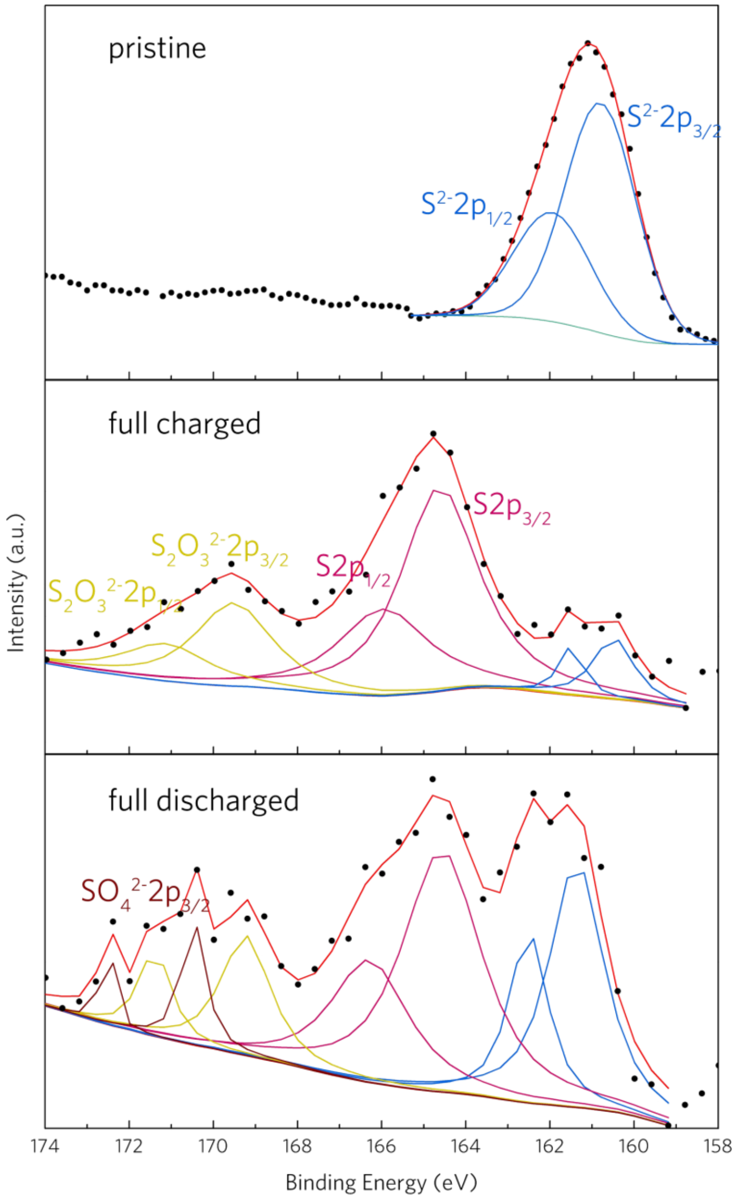

Supplementary Figure 16. S 2p XPS data of pristine, charged and discharged sample of $NaCr_{2/3}Ti_{1/3}S_2$. It can be seen that in pristine sample of $NaCr_{2/3}Ti_{1/3}S_2$ there are only peaks at 160.6 eV standing for S^{2-} 2p3 and after full charging the peaks of S_2^{2-} and higher valence like elemental sulfur and $S_2O_3^{2-}$ appear. After discharging process XPS peaks from elemental sulfur and $S_2O_3^{2}$ are observed, and the intensity of S^{2-} rises.

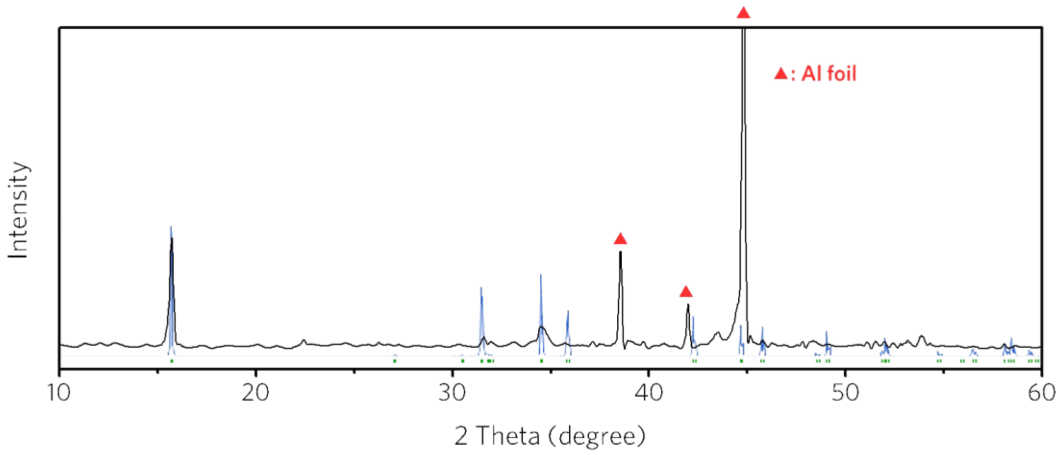

Supplementary Figure 17. XRD pattern of full charged $Na_0Cr_{2/3}Ti_{1/3}S_2$ comparing to the simulation results(a=3.37 Å, b=3.15 Å and c=16.94 Å, α=β=90°, γ=113°, ,according to the STEM results, with no Cr migration), (1 0 -2) peak at 31.7° and (1 0 4) peak at 34.5° are lower than theoretical value, showing that periodic structure had been changed as Cr migrated.

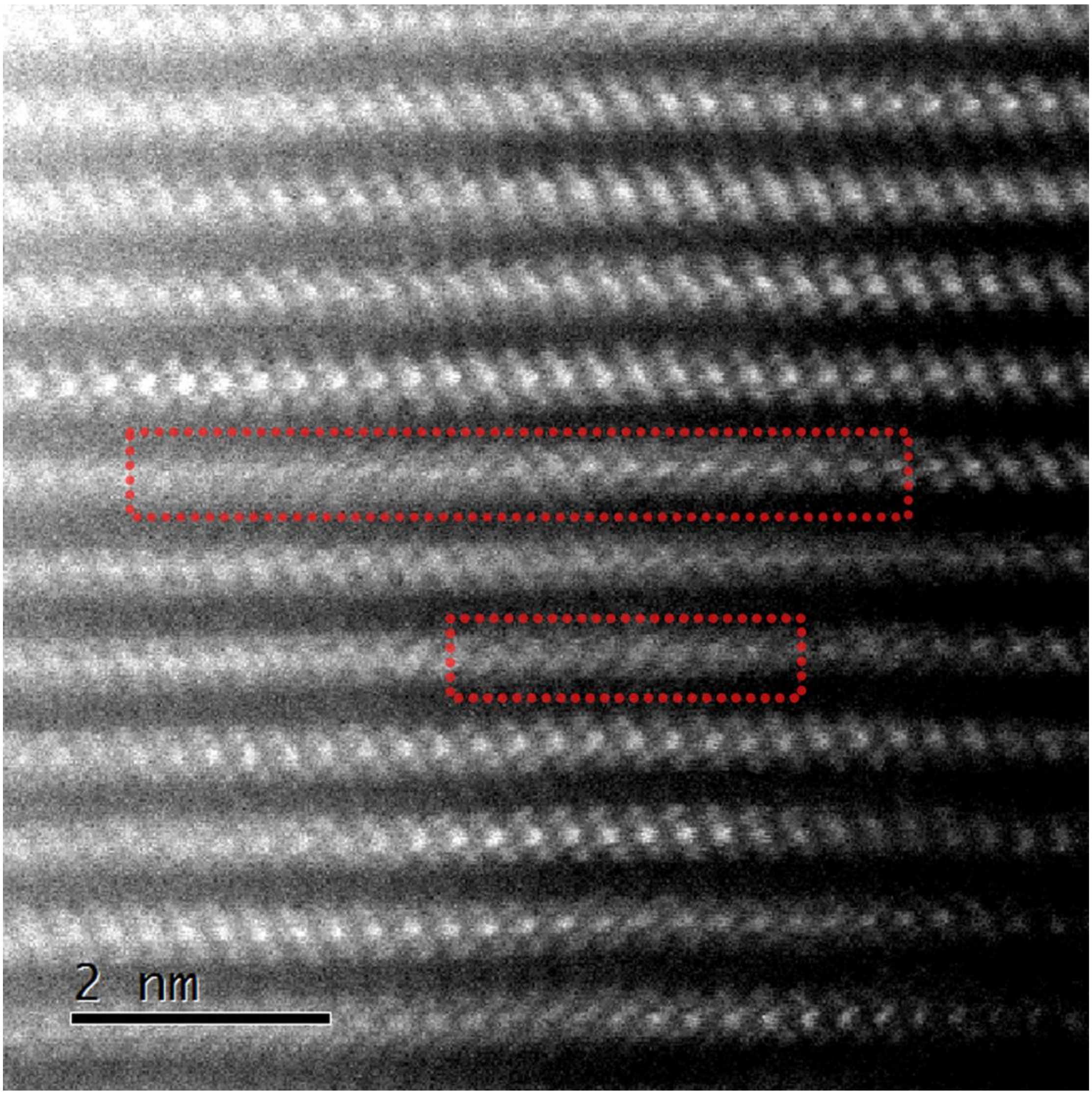

Supplementary Figure 18. STEM image of $NaCr_{2/3}Ti_{1/3}S_2$ electrode after 10 cycles, observed along a/b axis. Area marked by red box shows typical heterogeneous or amorphous phase, as a direct structural reason for capacity loss.

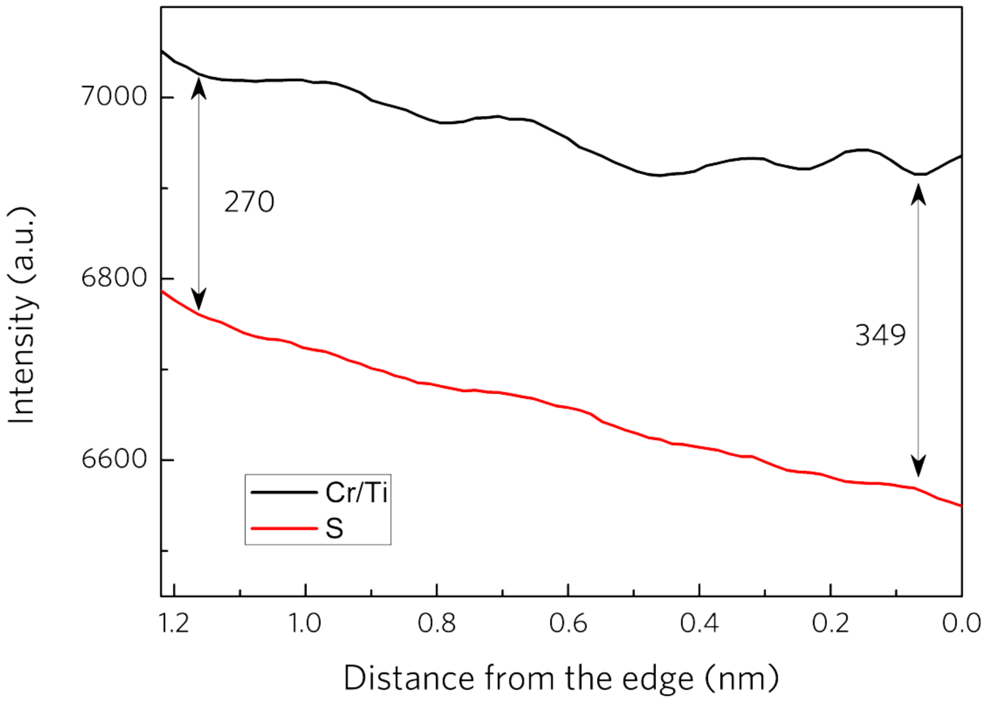

Supplementary Figure 19. STEM linear intensity profiles for the edge part of Supplementary Fig. 11. The difference in intensity between Cr/Ti layer and S layer increased from 270 on 1.2 nm from the edge to 349 on the site of the edge, which means that the crack might appear accompanied by sulfur loss. The mechanism of disproportionation of sulfide species near crack is proposed to be:

$(S_2)^{2-}$ (as $Cr_{2/3}Ti_{1/3}S_2$)$\rightarrow$ S + S^{2-} (Cr_2S_3,TiS_2 or $Cr_{2/3}Ti_{1/3}S_{5/3}$)

This equilibrium can move to the right with the precipitation of elemental sulfur near the crack, in the surface. This mechanism is raised to provide one possible origin of elemental sulfur. The main course of sulfur precipitation should come from the further oxidation of $(S_2)^{2-}$ in the charging process.

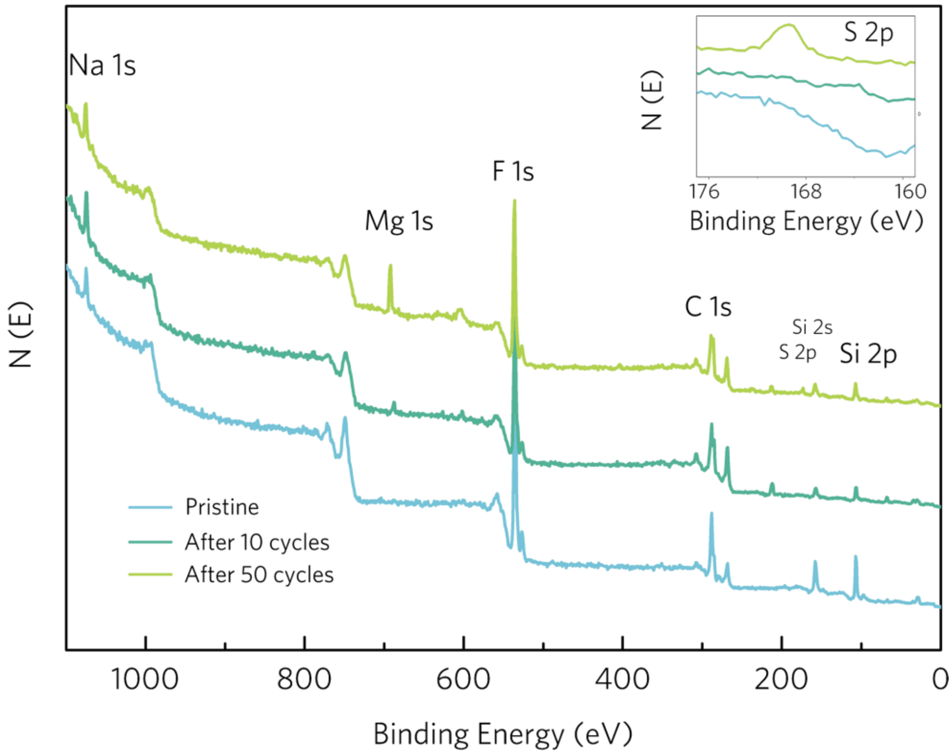

Supplementary Figure 20. XPS data of separator in $NaCr_{2/3}Ti_{1/3}S_2$ cell after 0, 10 and 50 cycles, respectively. There is no Ti or Cr peak found in the full spectrum, verifying the existence of sulfur is not owing to the deposition or dropping of $NaCr_{2/3}Ti_{1/3}S_2$.

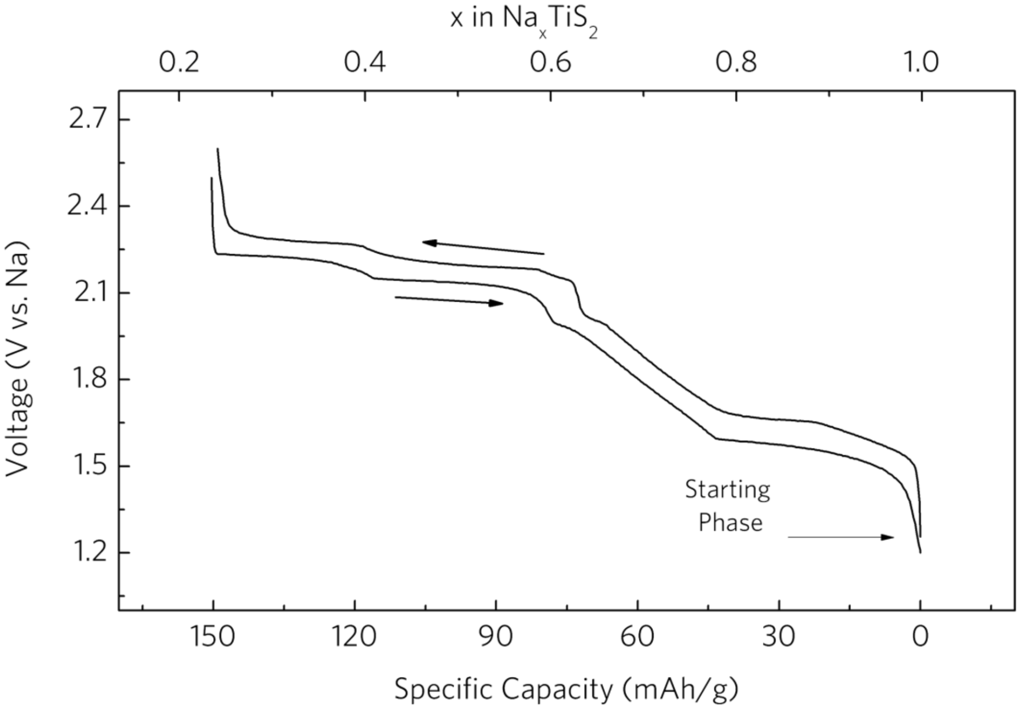

Supplementary Figure 21. The Voltage profile for the $NaTiS_2$ versus Na^+/Na at C/10 rate. Showing in $NaTiS_2$ cathode between 1.2-2.6 V, Ti can not be totally oxidized to Ti^{4+}, leading to a relatively low capacity of ~160 mAh/g comparing to theoretical capacity of 196 mAh/g, indicating that if charge transfer all comes from Ti, Ti^{3+} will be oxidized only to $Ti^{3.8+}$ eventually.

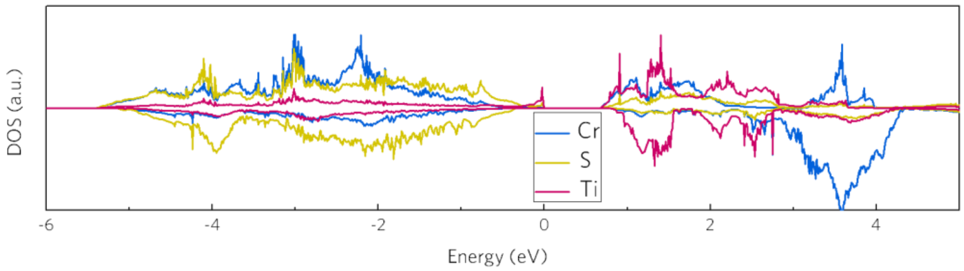

Supplementary Figure 22. DOS of $Na_{0.75}Cr_{2/3}Ti_{1/3}S_2$. It can be seen that in the DOS of $Na_{0.75}Cr_{2/3}Ti_{1/3}S_2$, when Ti is oxidized to $Ti^{3.75+}$, it still has occupied orbital at E_f, making it possible to be continuously oxidized to Ti^{4+}.

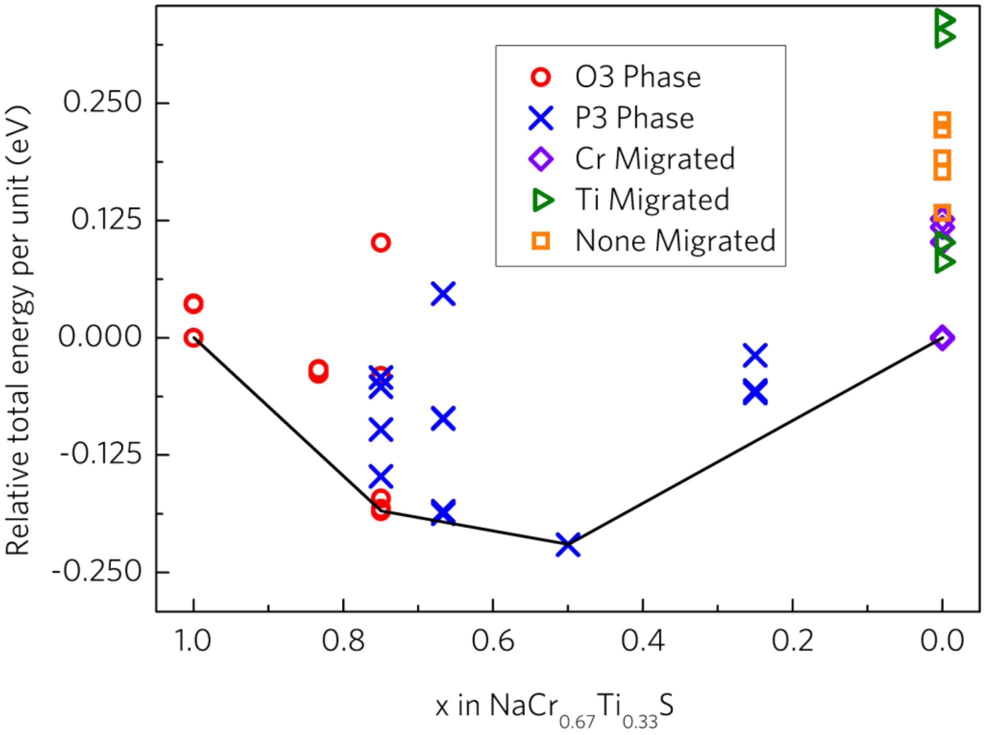

Supplementary Figure 23. Phase diagram of different Na content in $Cr_{2/3}Ti_{1/3}S_2$, showing a solid solution reaction for Na1-Na0.75, an O3-P3 phase transition at Na0.75, a solid solution reaction for Na0.75-Na0.5 and a P3-O1′ phase transition for Na0.5-Na0. The total energy is normalized to $Na_xCr_{2/3}Ti_{1/3}S_2$, a unit consisting of x Na atom, 0.67 Cr atom, 0.33 Ti atom and two S atoms.

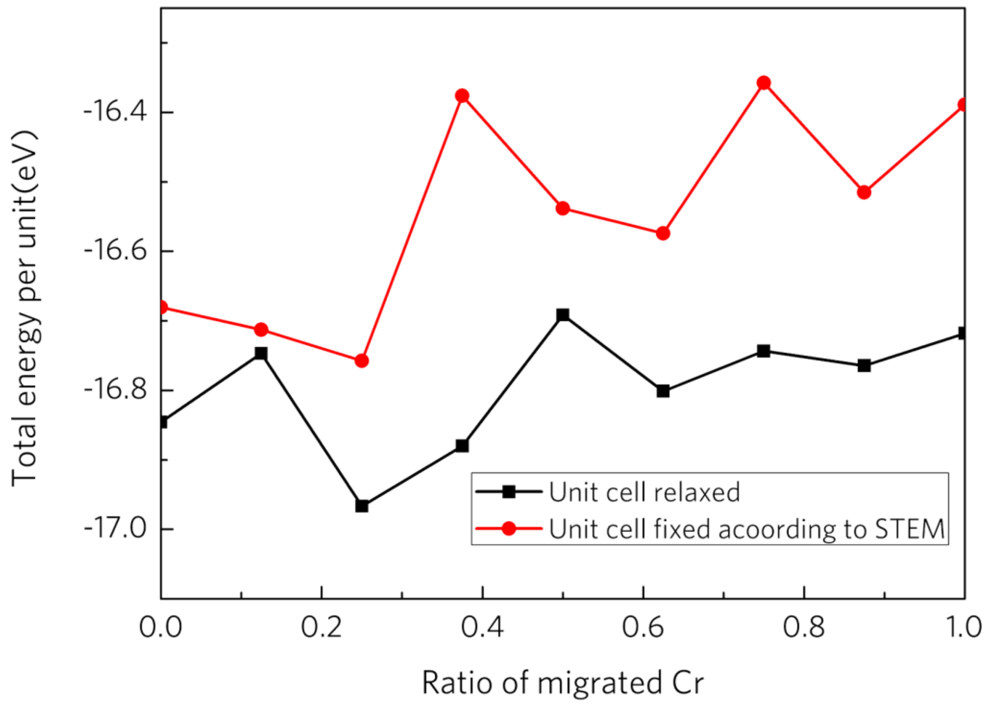

Supplementary Figure 24. Total energy after different ratio of Cr migrated to Na vacancies in $Na_0Cr_{2/3}Ti_{1/3}S_2$, with and without cell parameter constraint. The total energy is normalized to $Cr_{2/3}Ti_{1/3}S_2$, a unit consisting of 0.67 Cr atom, 0.33 Ti atom and two S atoms. In the figure only the lowest energy was showed, for example, in the case of 25% Cr migrated, the situation with lowest energy is two neighboring Cr migrated to the same next layer.

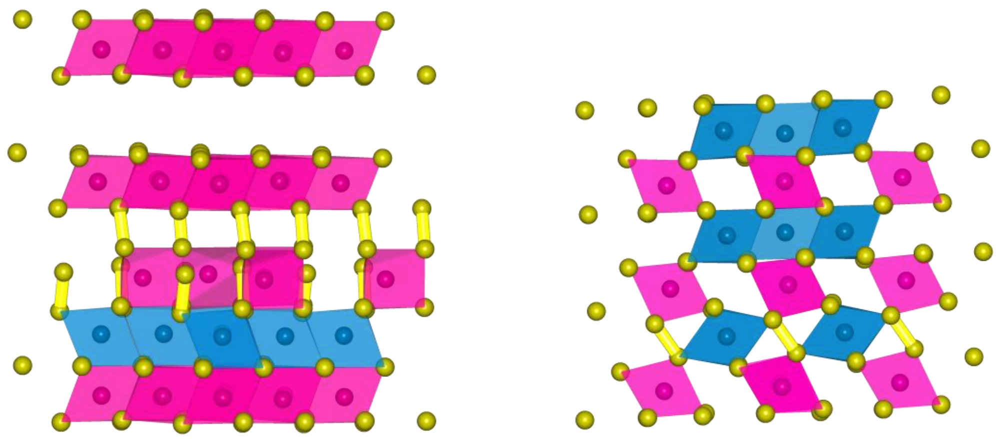

Supplementary Figure 25. Crystal structures exhibiting the migration of Cr and formation of S-S dimers for left, 2 Cr (25 %) migrated in 2 × 2 × 1 cell ($Cr_8Ti_4S_{24}$) and right 4 Cr (50 %) migrated in 2 × 2 × 1 cell ($Cr_8Ti_4S_{24}$), red octahedrons stand for not migrated Cr/Ti and blue octahedrons for migrated Cr, yellow balls for S and yellow sticks for S-S dimer. When the ratio of Cr migration is 25%, 1/3 of S formed S-S dimers (Fig. S21 left). When the ratio of Cr migration is 50%, 1/6 of S formed S-S dimers (Fig. S21 right).

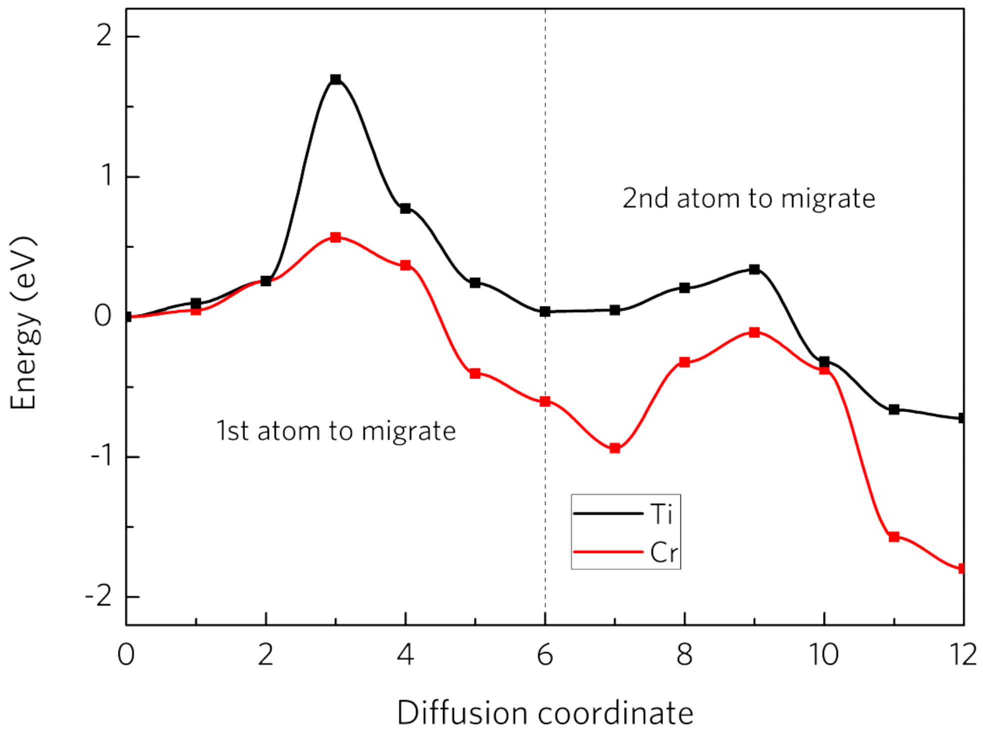

Supplementary Figure 26. Diffusion barriers of Cr and Ti for first two atoms to migrate in a 2 × 2 × 1 cell ($Cr_8Ti_4S_{24}$), The energy barrier is represented as total energy variation of the whole 2 × 2 × 1 cell. In Supplementary Fig. 17 and its corresponding analysis it can be concluded that in a 2 × 2 × 1 cell, the final structure with lowest total energy is a structure with two neighboring Cr or Ti migrated to the same next layer. The diffusion path is that the first Cr/Ti moves near directly along c axis towards next layer, and then the second. We have tried o-t-o path too but Cr/Ti seems not stable at tetrahedron in the presence of S-S dimer, the energy is higher than o-o path.

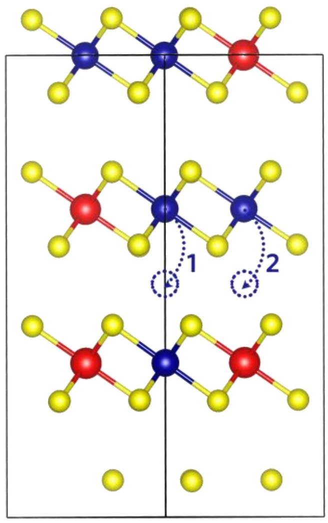

Supplementary Figure 27. Diffusion paths of Cr ions.

Supplementary Tables

Supplementary Table 1. EDS element content analysis results of the as prepared material.

Element	Series	Unnormalized Content (weight, %)	Content normalized (weight, %)	Content (atomic, %)	Content error (%)
Sodium	K-series	19.97	20.38	29.60	1.3
Sulfur	K-series	45.38	46.33	48.24	1.7
Chromium	K-series	18.69	19.08	12.25	0.8
Titanium	K-series	13.91	14.20	9.90	0.6

Supplementary Table 2. Summary of various polysulfide electrodes materials for lithium or sodium ion batteries.

Materials	Electrochemical window (V)	Capacity (mAh g^{-1})	Cycling stability	References
Bulk $NaCr_{2/3}Ti_{1/3}S_2$	1.4-3.3	186	52.2 % (360 cycles)	This work
$Na_{15}Sn_4/Na_3PS_4$ glass-ceramic/a-TiS_3.	1.2-2.6	300	33 % (10 cycles)	*1*
$Na_{15}Sn_4/Na_3PS_4$ glass-ceramic/a-TiS_3 with acetylene black	1.2-2.6	350	85.7 % (5 cycles)	*1*
FeS_2+Li_2S blended powder	1.0-3.0	650	26 % (15 cycles)	*2*
FeS_2-Li_2S composite	1.0-3.0	790	64 % (15 cycles)	*2*
VS_4/rGO composite versus Na^+/Na	0.01–2.2	450.4	53.5 % (50 cycles)	*3*
VS_4/rGO composite versus Li^+/Li	0.01–3.0	1669	57.2% (100 cycles)	*4*
a-TiS_4 versus Li^+/Li	1.6-3.0	609	67.1% (20 cycles)	*5*

Supplementary Table 3. Structural parameters and atomic positions of as-prepared $NaCr_{2/3}Ti_{1/3}S_2$ deducted from Rietveld refinement of synchrotron data.

$NaCr_{2/3}Ti_{1/3}S_2$						
Space group *R – 3 m* Rwp=16.95％ Rp=11.41％ χ2 = 1.733						
a = 3.554624(20) Å *c* = 19.58132(18) Å *V* = 214.2690(30) $Å^3$						
Atom	Wyckoff position	*x/a*	*y/b*	*z/c*	Occupancy	Uiso
Cr1	3a	0.0	0.0	0.0	0.652	0.00101(12)
Ti2	3a	0.0	0.0	0.	0.348	0.0040(14)
S3	6c	0	0	0.26519(8)	1.000	0.0032(8)
Na4	3b	0.0	0.0	0.5	0.973	0.00105(9)

Supplementary Table 4. Structural parameters and atomic positions of $Na_{0.66}Cr_{2/3}Ti_{1/3}S_2$ deducted from Rietveld Refinement

$Na_{0.66}Cr_{2/3}Ti_{1/3}S_2$

Space group *R* 3 *m* Rwp=6.45％ Rp=5.02％ χ2 = 1.208

a = 3.4042(5) Å *c* = 20.885(4) Å *V* = 209.60(6) $Å^3$

Atom	Wyckoff position	*x/a*	*y/b*	*z/c*	Occupancy	Uiso
Cr1	3a	0.0	0.0	0.0050(22)	0.652	0.0047(16)
Ti2	3a	0.0	0.0	0.0050(34).	0.348	0.024(10)
S3	3a	0	0	0.4049(18)	1.000	0.0124(33)
S4	9b	1/3	2/3	0.2814(12)	1.000	0.001(28)
Na5	3a	0.0	0.0	0.1704(27)	0.65(5)	0.09(4)

Supplementary Table 5. Stoichiometry of as-prepared $NaCr_{2/3}Ti_{1/3}S_2$ determined from ICP analysis with standard deviation (SD) and relative standard deviation (RSD) factors.

Composition from ICP			
Atom	Na	Cr	Ti
Content	0.973	0.652	0.348
SD	0.011	0.007	0.005
RSD (%)	1.1	1.1	1.4

Supplementary Table 6. Stoichiometry of full charged $Na_0Cr_{2/3}Ti_{1/3}S_2$ determined from ICP analysis.

Composition from ICP			
Atom	Na	Cr	Ti
Content	0.119	0.659	0.341
SD	0.004	0.009	0.005
RSD (%)	3.3	1.4	1.5

Supplementary Table 7. Stoichiometry of full discharged $NaCr_{2/3}Ti_{1/3}S_2$ determined from ICP analysis.

Composition from ICP			
Atom	Na	Cr	Ti
Content	1.041	0.662	0.338
SD	0.009	0.011	0.006
RSD (%)	0.9	1.7	1.8

Supplementary References

1 Tanibata, N., Matsuyama, T., Hayashi, A. & Tatsumisago, M. All-solid-state sodium batteries using amorphous TiS3 electrode with high capacity. *Journal of Power Sources* **275**, 284-287, (2015).

2 Takeuchi, T. *et al.* Improvement of Cycle Capability of FeS2 Positive Electrode by Forming Composites with Li2S for Ambient Temperature Lithium Batteries. *Journal of the Electrochemical Society* **159**, A75-A84 (2012).

3 Sun, R. M. *et al.* Vanadium Sulfide on Reduced Graphene Oxide Layer as a Promising Anode for Sodium Ion Battery. *Acs Appl Mater Inter* **7**, 20902-20908 (2015).

4 Rout, C. S. *et al.* Synthesis and characterization of patronite form of vanadium sulfide on graphitic layer. *J Am Chem Soc* **135**, 8720-8725, (2013).

5 Sakuda, A. *et al.* Amorphous Metal Polysulfides: Electrode Materials with Unique Insertion/Extraction Reactions. *Journal of the American Chemical Society* **139**, 8796-8799 (2017).
